# The impact of cesarean section on neonatal outcomes at a university-based tertiary hospital in Jordan

**DOI:** 10.1186/s12884-020-03027-2

**Published:** 2020-06-01

**Authors:** Wasim Khasawneh, Nail Obeidat, Dawood Yusef, Jomana W. Alsulaiman

**Affiliations:** 1 Department of Pediatrics and Neonatology, Faculty of Medicine, Jordan University of Science and Technology, Irbid, Jordan; 2 Department of Obstetrics and Gynecology, Faculty of Medicine, Jordan University of Science and Technology, Irbid, Jordan; 3 Department of Pediatrics, Medical school, Yarmouk University, Irbid, Jordan

**Keywords:** Cesarean section, Neonates, Neonatal ICU, Neonatal outcomes

## Abstract

**Background:**

Over the past two decades, there has been a steady rise in the rate of Cesarean section delivery globally. As a result, short-term and long-term maternal and neonatal complications are rising. The objective of this study is to determine the rate and indications for Cesarean section at King Abdullah University Hospital (KAUH) in Jordan and to assess the resulting neonatal outcomes.

**Methods:**

A retrospective chart review was conducted for all women and neonates delivered by Cesarean section during the period January 2016 to July 2017 at KAUH tertiary academic center. Collected data include demographic characteristics, indication for delivery, and neonatal outcomes such as NICU admission, respiratory complications, sepsis, mortality, and length of hospitalization.

**Results:**

Two thousand five hundred ninety-five Cesarean section deliveries were performed over 18 months representing a rate of 50.5% of all deliveries. Sixty percent were scheduled procedures. Seventy-two percent were performed at full term gestation. The most common indication was previously scarred uterus (42.8%) followed by fetal distress (15.5%). The rate of admission to the neonatal ICU was 30% (800/2595). After multilogistic conditional regression analysis, the factors associated with increased risk of neonatal ICU admission were found to include grandmultiparity (Adjusted OR 1.46), gestational diabetes (Adjusted OR 1.92), maternal employment (Adjusted OR 1.84), prolonged rupture of membranes (Adjusted OR 5), fetal distress (Adjusted OR 1.84), prematurity (Adjusted OR 43.78), low birth weight (Adjusted OR 42), high order multiple gestation (Adjusted OR 9.58) and low 5-min APGAR score (Adjusted OR 10). Among the babies electively delivered at early term (37–38.6 weeks), 16% were admitted to the NICU for a median length of stay of 4 days (IQR 2, 8). The most common diagnoses for admitted term neonates were transient tachypnea of newborns and respiratory distress syndrome.

**Conclusions:**

CS deliveries account for more than half the number of deliveries at our institution and almost one third of the delivered babies are admitted to the NICU. Together with the resulting maternal and neonatal consequences, this carries a major burden on the newborns, health care facilities, and involved families. Local strategies and policies should be established and implemented to improve the outcome of births.

## Background

Cesarean section (CS) is the most commonly performed surgical procedure in obstetrics [1]. In the past, CS was performed for pure obstetric indications where vaginal delivery carries risks on the mother and the baby. The World Health Organization (WHO) has repeatedly reported any population-based rate of CS delivery should not exceed 15% [2]. More recently, WHO stated that the optimal rate for CS is unknown and emphasized that this procedure should be ideally performed when medically indicated [3]. However, with the advance in anesthesia and postoperative care over the past one to two decades, the rate of cesarean section rose all across the world with a variable reported rate of 15–40% amongst different nations and institutions [4, 5]. Although not well understood, multiple factors have contributed to this uptrend. Maternal indications for CS include previous CS delivery, antepartum hemorrhage, uncontrolled hypertension, and failure to progress of labor [6]. Fetal indications include fetal distress, malpresentation, cephalopelvic disproportion and certain major congenital anomalies [6]. The goal of CS delivery is to avoid the complications that might develop after vaginal delivery. However, this major surgery is not without significant impact on maternal and fetal/ neonatal outcomes. Previous studies have reported an increase in maternal mortality up to three times with CS delivery [1]. Similarly, the rate of maternal complications increases two and five folds after elective and emergency CS respectively [1]. Maternal complications include the increased risk of postpartum hemorrhage, risk of hysterectomy, infection and deep venous thrombosis besides longer hospital stay and increased risk in subsequent pregnancies. Data about the effect of increasing CS rate on reducing neonatal complications is conflicting between different centers [7–9]. Besides the increase in mortality rate, fetal and neonatal complications include the increased risk for neonatal ICU admission, respiratory morbidities and mother-infant separation with all its consequences [10]. In 2016, Kupari et al. from Finland concluded that the increase in CS rate does not lower the incidence of neonatal asphyxia. Rather, the rate of NICU admissions was higher after CS deliveries in their review [11]. More recently, studies have supported the use of antenatal steroids to reduce respiratory morbidities among babies born by elective CS at late preterm and term gestation [12–14].

In Jordan, few studies have been published over the past decade indicating national increase in the rate of CS delivery [15, 16]. However, neither the rate of CS delivery nor the resultant maternal and fetal/ neonatal outcomes have been specifically studied in the past at any of the academic hospitals where resident trainees are more involved, so we decided to conduct this project to shed light on our outcomes as a university based tertiary care center.

## Methods

The objective of this study is to determine the rate of CS delivery at KAUH, review the indications for CS, and assess neonatal outcomes including NICU admission rate, respiratory morbidities, sepsis, mortality, and length of hospitalization.

A retrospective chart review was conducted for all CS deliveries at KAUH in Jordan in the period January 2016 to July 2017. KAUH is a university-based tertiary care center affiliated with Jordan University of Science and Technology. It is located in the city of Irbid and provides health service to nearly two million of the Jordanian population. Most of our patients have governmental and employer-based health insurance. The number of annual deliveries approaches 3500. The obstetric service is operated by ten full-time consultant obstetricians with around 30 postgraduate training residents. Besides the routine obstetric service offered to patients who have medical insurance coverage through KAUH, our institution is the main referral center for high risk pregnancies in Northern Jordan. High risk pregnancies are mostly followed by three qualified maternal-fetal medicine specialists. In our center, despite lacking a written policy regarding the indication and timing for elective CS delivery, there is a general consensus among all obstetricians to follow the ACOG recommendations. However, the consultant obstetricians are not in-house all the time and the service is run by high-level, well-trained postgraduate residents in the afterhours while the consultants are required to be available within a short distance from the hospital. Our institution is the main center for in-Vitro Fertilization (IVF) in Northern Jordan and is considered the major referral center for all IVF pregnancies in the region.

The GA of our participants was determined as documented in the electronic charts of the pregnant women and the neonates after delivery. Most of the women included in our analysis had their booking visit early in gestation and the gestational age is estimated based on early ultrasound and/or last menstrual period. After birth, all babies are clinically assessed using Ballard maturity scale. The discrepancy between antenatal calculation and postnatal assessment is minimal in the majority of cases.

After delivery, well-looking late preterm and full term newborns are usually admitted to the well-baby nursery. Babies with mild respiratory distress are given a 2 to 3-h chance for transitioning in the well-baby unit as well. Among the indications for NICU admission are all < 35-week preterm babies, respiratory distress requiring any respiratory support beyond transitioning, cases that require intravenous antibiotics based on risk assessment for sepsis, and cases with major dysmorphism or suspected surgical problems. Also, newborns in the well-baby nursery who require escalation of support due to any change in their clinical exam, respiratory status, feeding issues or jaundice are transferred to the NICU.

The list of all CS deliveries during the study period was extracted from the hospital electronic database after an official approval was obtained from the hospital administration. An Institutional Review Board (IRB number 388–2017) approval was obtained from Jordan University of Science and Technology. Patient’s consent was waived. Data collected includes maternal demographics, past obstetric history, associated medical problems, type of CS (elective vs emergency), indication for delivery, type of anesthesia, newborn outcomes including birth weight, 5-min Apgar score, NICU admission, respiratory status, the need for respiratory support, rate of sepsis and length of stay.

Data was collected by well-trained postgraduate residents under the supervision of a consultant neonatologist and a consultant obstetrician. Data was collected in an excel sheet and completed for more than 99% of the included women and their newborns. Neonatal outcomes were based on the diagnoses assigned by the treating neonatologist as documented in the electronic medical records.

### Statistical analysis

SPSS version 22 was used for data management and analyses (IBM Corp., Armonk, N.Y., USA). Frequency distribution (numbers and percentages) and mean (SD) were produced for all variables as appropriate. At the bivariate level, distribution of each independent variable by the outcome of NICU admission was assessed using X^2^ test or t-test, as appropriate, along with *P*-values. Variables that were associated with admission were then included in a backward conditional logistic regression level (entry level: 0.05, removal level: 0.2). Variables identified by the regression model were presented using Adjusted Odds Ratios and 95% Confidence Intervals (AOR, 95% C.I.). Alpha level was set at 0.05.

## Results

During the study period, 2595 CS deliveries were performed at KAUH. This represents 50.5% of all deliveries. Of the CS deliveries, 60% were electively planned procedures among term and late preterm pregnancies and 40% performed as emergency deliveries. Table 1 shows the maternal and neonatal characteristics of the studied population. Seventy-two percent of CS deliveries were performed at full term gestation and 13% of cases were multiple gestation pregnancies. General anesthesia was used in almost one third of the cases.

**Table 1:** Maternal and neonatal characteristics

		Number (*N* = 2595)	Percent
**Maternal**			
Age (years)	< 21	57	2.2%
	21 to 35	1930	74.4%
	> 35	608	23.4%
Parity	1	643	24.8%
	2	1172	45.2%
	≥ 3	780	30.1%
IVF	No	2361	91.0%
	Yes	234	9.0%
Preeclampsia	No	2541	97.9%
	Yes	54	2.1%
Gestational DM	No	2526	97.3%
	Yes	69	2.7%
Previous CS	No	1075	41.4%
	Yes	1520	58.6%
Employed	No	801	30.9%
	Yes	1794	69.1%
AN steroids	No	2109	81.3%
	Yes	486	18.7%
Induction trial	No	2339	90.1%
	Yes	256	9.9%
PROM	No	2305	88.8%
	Yes	290	11.2%
Elective	No	1038	40%
	Yes	1557	60%
Anesthesia	General	805	31%
	Spinal	1790	69%
**Neonatal**			
Gestational age (weeks)	< 35	259	10%
	35 to 36	471	18.2%
	≥ 37	1865	71.9%
Birth Weight (grams)	Mean (SD)	2910 (630)	–
	≤ 1500	90	3.5%
	1501–2500	496	19.1%
	> 2500	2009	77.4%
Gender	F	1235	47.6%
	M	1360	52.4%
Multiples	Singleton	2269	87.4%
	Twins	258	9.9%
	Triplets	57	2.2%
	Quadruplets	11	0.4%
5-min APGAR	< 7	66	2.5%
	≥ 7	2529	97.5%

*IVF* In Vitro Fertilization, *DM* Diabetes Mellitus, *CS* Cesarean section, *PROM* Prolonged rupture of membranes, *(SD)* Standard deviation, *F* Female, *M* Male

The main indication for CS delivery was previous CS delivery (43%) followed by fetal intolerance to labor (15.5%), maternal request (14.9%) and failure to progress (6%). Nearly 10% of CS deliveries were performed after failure of labor induction trial. Table 2.

**Table 2:** Indications for CS

Indication	Number	Percentage
Previous CS/ No VBAC trial	1108	42.8%
Fetal distress	402	15.5%
Maternal request	386	14.9%
Breech presentation	199	7.7%
Failure to progress	164	6.3%
Multiple gestation	157	6.1%
Preeclampsia	90	3.5%
Antepartum hemorrhage	83	3.2%
others	2	0.1%

*CS* Cesarean Section, *VBAC* Vaginal birth after cesarean section

A total of 800 neonates were admitted to the neonatal Intensive Care Unit (NICU) following CS delivery. Of those, 43% (346) were born at full term gestation. The rate of NICU admission was 23% among the elective procedures compared with 43% in the emergency CS deliveries.

The factors associated with increasing risk of NICU admission among CS delivery, as reported in Table 3**,** were then included in a backward conditional logistic regression model (entry level: 0.05, removal level: 0.2). With this logistic regression model, the following factors were found to be significantly associated with increased rate of NICU admission.

**Table 3:** Risk factors for NICU admission following CS delivery

	NICU admission n (%)			Adjusted effect		
	No	Yes	*P* value	AOR	95% C.I	
**Age (years)**						
< 21	36 (60)	23 (40)	0.287			
21 to 35	1339 (69)	589 (31)		–		
> 35	420 (69)	188 (31)				
**Parity**						
1	391 (61)	254 (39)	0.000	Ref		
2	865 (74)	305 (26)		1.23	0.86	1.74
≥ 3	539 (69)	241 (31)		1.46	1.03	2.07
**IVF**						
No	1693 (72)	667 (28)	0.000	–		
Yes	102 (43)	133 (57)				
**Preeclampsia**						
No	1770 (70)	771 (30)	0.000	–		
Yes	25 (46)	29 (54)				
**Gestational DM**						
No	1758 (70)	768 (30)	0.004	Ref		
Yes	37 (54)	32 (46)		1.92	1.05	3.53
**Previous CS**						
No	634 (60)	441 (40)	0.000	Ref		
Yes	1161 (76)	358 (24)		0.70	0.49	0.99
**Employed**						
No	596 (74)	206 (26)	0.000	Ref		
Yes	1199 (67)	594 (33)		1.84	1.44	2.34
**Induction trial**						
No	1617 (70)	720 (30)	0.472	Ref		
Yes	178 (69)	80 (31)		0.62	0.41	0.93
**PROM**						
No	1689 (74)	613 (26)	0.000	Ref		
Yes	106 (36)	187 (64)		5.0	3.6	6.9
**Elective**						
No	595 (57)	443 (43)	0.000	–		
Yes	1200 (77)	357 (23)				
**G age (weeks)**						
< 35	11 (4)	249 (96)	0.000	43.78	21.21	90.37
35 to 36	267 (56)	205 (44)		2.34	1.79	3.07
≥ 37	1517 (81)	346 (19)		Ref		
**Birth weight**						
≤ 1500	5 (5)	90 (95)	0.000	42.01	19.08	88.44
1501–2500	211 (43)	285 (57)		5	4.1	6.2
> 2500	1579 (79)	425 (21)		Ref		
**Gender**						
F	859 (69)	377 (31)	0.383	–		
M	936 (69)	423 (31)				
**Multiples**						
Singleton	1661 (73)	605 (27)	0.000	Ref		
Twins	133 (51)	128 (49)		0.58	0.35	0.96
Triplets	1 (2)	56 (98)		9.58	1.12	82.21
Quadruplets	0 (0)	11 (100)				
**5-min APGAR**						
< 7	7 (11)	59 (89)	0.000	10.01	4.07	24.60
≥ 7	1784 (71)	741 (29)		Ref		

*NICU* Neonatal intensive care unit, *IVF* In Vitro Fertilization, *DM* Diabetes Mellitus, *CS* Cesarean section, *PROM* Prolonged rupture of membranes, *G age* Gestational age, *M* Male, *F* Female, *AOR* Adjusted Odds Ratio, *C.I* Confidence Interval

### Maternal factors

- Parity status: Increased rate among grand multiparous (≥ 3) mothers (AOR 1.46)
- Maternal morbidities: Increased rate among mothers with gestational diabetes (AOR 1.92).
- First time CS delivery (AOR 1.45)
- Mothers with prolonged rupture of membranes before delivery with clinical suspicion of chorioamnionitis (AOR 5.0).
- Maternal employment (AOR 1.84)

### Fetal/ neonatal factors

- Emergency procedures due to fetal distress (AOR 1.84).
- Prematurity (AOR 2.34 for 35–37 weeks and 43.78 for < 35 weeks).
- Low birth weight (AOR 42 for < 1500 g and 2.22 for 1500–2500 g)
- High order multiple gestations (≥ 3) (AOR 9.58).
- Low APGAR score at 5 min (AOR 10).

Table 4 shows the outcomes of the neonates admitted to the NICU. Among the neonates admitted to NICU, more than half required respiratory support for at least 24 h. Of the 459 admissions who required respiratory support in the form of continuous positive airway pressure (CPAP) or invasive mechanical ventilation for diagnosis of respiratory distress syndrome or transient tachypnea of newborn, 139 (30%) were term babies of whom two thirds (88/139) were born by elective planned CS between 37 and 38 6/7-week gestation. 126/800 (16%) received surfactant (24 term vs 122 preterm), 18% of NICU admissions were complicated by sepsis. The mortality rate was 5%.

**Table 4:** Neonatal outcomes of NICU admissions

		Number	Percent
Direct Admission	No	271	33.9%
	Yes	529	66.1%
TTN/ RDS	No	359	44.8%
	Yes	441	55.2%
CPAP	No	354	44.2%
	Yes	446	55.8%
Surfactant	No	674	84.5%
	Yes	126	15.5%
Sepsis	No	655	81.9%
	Yes	145	18.1%
Mortality	No	760	95.0%
	Yes	40	5.0%

*NICU* Neonatal intensive care unit, *TTN* Transient tachypnea of the newborn, *RDS* Respiratory distress syndrome, *CPAP* Continuous positive airway pressure

## Discussion

Our study demonstrates a high rate of CS delivery exceeding half of all the deliveries at our institution. Almost one third of the delivered neonates were admitted to the NICU. This high rate of CS and NICU admission could be related to the fact that KAUH is a referral center with a high rate of high-risk pregnancy referrals including multiple-gestation pregnancies.

Globally, there has been an uptrend in the rate of CS deliveries over the past two decades. Although considered extremely high, the rate of CS delivery in the present study is consistent with the rates reported from some other countries in the region. According to the WHO, the rates of CS in the East Mediterranean Region varies with high reported rates of 52, 48 and 46% in Egypt, Iran and Lebanon respectively, and low rates between 5 and 25% in some other countries [17]. Similarly, a recent report published in 2016 showed an increase in CS rate from 20 to 42% in Latin America and 14 to 25% in Europe [4]. In the US, the CDC reports about CS delivery have shown a national increase in CS rate over the past few years reaching as high as 38% in the southern states [18]. To better understand the uptrend in CS rate and delineate the variation between nations and facilities, the WHO proposed a complete perinatal classification system named as Robson classification that can be utilized as a standard tool in categorizing women at the time of delivery and allowing rate analysis and comparison between different nations and centers [19].

The reported rate of more than 50% in the present study had raised a strong alarm about the situation in our institution. This is actually a major public health concern that drives urgent discussions about establishing local and national policies to be among the top priorities for the health care providers and decision makers. Simultaneously, health care providers should strictly adhere to these policies to improve the outcome at local and national levels.

Among the factors that could have contributed to the global increase in the rate of CS procedures are the patients’ worries about the potential complications of vaginal delivery, the socio-cultural changes with improved maternal education and economic status increasing maternal demand for CS delivery, the increase in malpractice claims, and the limited provider education in utilizing technology to assess the risk of vaginal delivery mainly after induction of labor [15, 17, 20]. In a recently published report by Betran et al., the authors highlighted some of the interventions required to avoid unnecessary CS procedures. Examples include improving the obstetric care providers’ education and training regarding optimal vaginal birth, optimizing facility resources, and encouraging more education and counselling during antenatal care visits [3].

In Jordan, a national study published in 2017 reported a CS rate of 29% among different Jordanian hospitals [16]. In their study, Batieha et al. reported a higher rate of CS in teaching and private hospitals, they also reported higher rate of CS with previously scarred uterus and fetal distress. The neonatal mortality rate was also higher compared with vaginal births. Ten years earlier, the rate of CS was about 18% as reported by Department of Statistics (Jordan) and Macro International Inc. 2008 [21]. Factors that could possibly explain the higher rate in teaching and private hospitals include performing unindicated operations for the purpose of training the resident physicians or mainly for better financial gain.

The indications for CS deliveries among our patients are consistent with other international reports [4, 15, 22]. The main reported indication of CS in our study is a scared uterus. This was the main medically approved indication in the majority of other studies. This factor should draw the attention of all healthcare decision makers to review the indication of the first time CS and make sure it is medically indicated according to strict medical policies since this is the main determinant for repeat sections in the future especially in places where large family size is preferred and could be potentially limited with repeated CS deliveries.

The increase in maternal request for elective CS in the absence of any medical or obstetric indications has added to the rising rate of CS in multiple centers [23]. In our study, maternal request accounted for about 15% of the CS procedures. On the contrary, this has not been of a great concern in Canada and Switzerland where the obstetricians stick to the local guidelines and don’t perform CS solely upon maternal request [24]. More detailed counselling should be provided to expectant mothers about the short and long-term consequences of this procedure, obstetricians need to focus on medical indications during their discussion and guide the pregnant women in making their decision.

Multiple gestations are considered among the high risk pregnancies. Studies have shown that the increasing rate of IVF procedures and other reproductive interventions resulting in multiple gestations has also contributed to higher CS rate in tertiary centers [24]. The ACOG recommendations regarding a single embryo transfer and avoidance of multifetal gestation in such procedures should be strongly enforced to reduce the adverse maternal and neonatal outcomes [25].

Although emergency CS procedures are intended to lower maternal and neonatal mortality and morbidities, it is clear that CS delivery might be associated with negative short-term and long-term consequences. This has been reported in several studies across the world with prolonging postpartum pain, analgesic intake, and hospital stay as well as increasing rate of NICU admissions with all resulting social and financial burdens [24, 26, 27].

Our present study showed a rate of NICU admission after CS of about 30% for all groups and 19% among term births. This is compared to an overall admission rate of 20 and 11% respectively among all deliveries at our institution. Also, we reported a 23% rate following pre-labor elective procedures performed after 35 weeks. In a single maternity hospital in Ireland, Finn et al. reported a NICU admission rate of 22% among early term neonates born by elective CS at 37 weeks compared with 10% for those born at 39 weeks [8]. In our region, data is limited about the incidence of NICU admission following CS delivery. In a quietly similar setting in Saudi Arabia, the overall rate of NICU admission among term infants is 4.1%, the specific rate of admission following CS delivery was not reported. However, half of their admitted term neonates were delivered by CS and half of the CS deliveries were elective procedures [28]. The variation in the rate of NICU admission worldwide might be explained by the different admission criteria implemented by various hospitals. These factors are affected by the level of care provided at the local neonatal ICU’s, the presence of intermediate care units where neonates with less acuity are usually cared for, the type of obstetric population, and the presence of local hospital guidelines. Neonates born by CS are known to have a higher NICU admission rate when compared to those delivered by vaginal birth or vaginal birth after CS (VBAC) [9]. However, there has been an uptrend for NICU admission among more mature newborns especially those delivered by planned CS in the late preterm and early term categories (35–39 week gestation) [8, 29] In our cohort, 36% of the NICU admissions were born by a planned CS delivery after 35-week gestation. Term babies constitute 43% of all NICU admissions (346 out of 800), of whom nearly half (159/346) were born electively between 37 and 38.6 weeks. The main indication for admission of term babies is for respiratory support secondary to delayed transitioning and the median length of stay among this group of babies is 4 days (IQR 2, 8 days). By further exploration of the adverse outcomes of pre-labor CS delivery, we found that about one out of 4 babies delivered by a planned scheduled CS after 35 weeks’ gestation was admitted to the NICU. We had also noticed that 80% (988/1227) of planned CS deliveries among term babies were performed between 37 and 38.6 weeks and the rate of NICU admission among this group in particular was 16% (159/988). Same finding of high rate of NICU admission among early term CS deliveries was reported by Wilmink from Netherlands [30] which emphasizes the importance of avoiding elective CS before 39 weeks.

Regarding neonatal outcomes, our findings are consistent with several other studies reporting an increase in respiratory morbidity among term babies born by planned CS [31, 32]. This can be explained by the fact that fetal lung fluid clearance is delayed or impaired after planned CS deliveries without going through labor first [33]. In our cohort, almost one third of the neonates admitted to the NICU were given a 2 to 3-h chance of transitioning in the newborn nursery before getting transferred to the NICU for respiratory support.

Therefore, it is clear that the respiratory outcomes of term neonates are not only reported to be worse after CS delivery when compared to vaginal birth. Rather, the exact gestational age plays an important role on the outcome of these babies. Studies have reported a better outcome for neonates delivered by elective CS if the procedure is performed after completed 39-week gestation [34, 35]. Salemi et al. found that the outcome of early term delivery is significantly worse among neonates born by elective CS when compared to those born after labor induction [36]. This concludes that adverse neonatal respiratory outcomes could be potentially decreased not only by avoiding CS delivery but also by the advancing GA even in the full term category. The compliance with the ACOG recommendations regarding avoiding early term delivery should be strongly encouraged to avoid such unwanted complications [37]. Although delayed transitioning and TTN are considered benign, the social and financial burdens of having babies admitted to the NICU for few days should not be underestimated [34, 35].

Another downside of CS delivery that is not highlighted by most studies is the decline in breastfeeding rate in those neonates compared with vaginal birth. In 2017, we have published a study about predictors and barriers to exclusive breastfeeding in Jordan and found a strong negative association between breastfeeding trends and CS delivery [38].

Although we hereby reported data from a single center which might not reflect the actual trend in the whole country, we believe that this is a point of strength as this is the first reported data about CS delivery and neonatal outcomes in an academic referral institution from Jordan where postgraduate residents play a major role in obstetric service. Up to our knowledge, this is also the first analysis reporting NICU admission rate of different GA categories among infants delivered by CS in the region. The main limitation of our study is the retrospective chart review design which makes it difficult to accurately infer conclusions. Also, the reasons for performing CS procedures were primarily provided by the on-call physician who is likely to provide reasonable justifications for performing CS although this may not reflect the actual indications. By conducting analysis of larger cohorts from different hospital settings, it is expected to reach more generalized conclusions at a national level. Application of Robson classification system in different health centers would help more in such analysis and comparisons [19].

## Conclusions

In conclusion, the rate of CS delivery is trending up and this has contributed to the increase in NICU admissions resulting in significant medical, social and financial impacts in the involved families and health care facilities. One of the main reasons for the increase in NICU admission rate is performing elective procedures at late preterm and early term gestation. Major hospitals and academic institutions should implement local strategies and policies, and strictly emphasize on following them to avoid any bias in selecting the mode of delivery in order to improve the outcome of births. Application of monitoring pathways should be established at the level of national health agencies. The compliance with the ACOG recommendations regarding abstinence from multi-embryo transfer in IVF procedures should be strongly encouraged to minimize maternal and neonatal adverse outcomes. Similarly, adhering to the recommendations of WHO, ACOG and other international obstetric organizations regarding avoiding elective CS procedures before 39-week gestation is key factor to avoid preventable causes of respiratory morbidities and reduce the rate of NICU admissions among this group of neonates.

## Data Availability

The datasets used and/or analyzed during the current study are available from the corresponding author on reasonable request.

## Abbreviations

KAUH: King Abdullah University Hospital
NICU: Neonatal Intensive Care Unit
CS: Cesarean Section
WHO: World Health Organization
ACOG: American College of Obstetrics and Gynecology
RDS: Respiratory Distress Syndrome
TTN: Transient Tachypnea of Newborns
IVF: In Vitro Fertilization
VBAC: Vaginal Birth After Cesarean section
